# Unraveling the impact of chili pepper geographical origin on beef tallow system: Integrated flavoromics and physicochemical profiling by HS-GC-IMS, GC × GC–MS, and *E*-nose

**DOI:** 10.1016/j.fochx.2026.104693

**Published:** 2026-10-26

**Authors:** Min Xu, Daitian Yan, Xiaoxiao Chen, Linjie Hu, Lan Li, Ling Li, Hongbin Lin, Qin Xiang, Wenwen Hou, Ping Liu

**Affiliations:** a College of Food and Bioengineering, Xihua University, Chengdu 610039, China; b Sichuan Chuanjiao Seed Technology Co., Ltd. Zigong 611732, China; c China Agricultural University-Sichuan Advanced Agricultural & Industrial Institute, China

**Keywords:** Chili pepper, Geographical origins, Volatile compound, PLS-DA, Entropy weight-TOPSIS method

## Abstract

The geographical origin of chili peppers influences the sensory quality of Sichuan hotpot in beef tallow systems, yet systematic evaluations remain limited. Chili peppers from six Chinese origins (WS, KS, JT, YM, AKS, and AM) were evaluated after thermal processing with beef tallow. Color parameters, capsaicin content, and volatile profiles were analyzed using GC × GC–MS, GC-IMS, and *E*-nose, and 18 compounds with rOAV ≥1 were screened as key aroma contributors. PLS-DA clearly differentiated the six samples by origin. Entropy weight–TOPSIS ranking was highly indicator-dependent: excluding volatiles detected in fewer than three origins changed the ranking from YM > WS > KS > JT > AKS > AM to WS > AKS > YM > KS > JT > AM. These findings show that origin-associated quality differences in chili–beef tallow systems are multidimensional and detectable under standardized processing, while composite rankings based on sparse volatile data should be interpreted with caution.

## Highlights

• Origin-dependent flavor of chili with beef tallow were systematically revealed.
• GC × GC–MS, GC-IMS, and E-nose effectively discriminated six chili pepper origins.
• A total of 82 volatiles (GC × GC–MS) were identified, with 18 key odorants.
• Entropy-weighted TOPSIS ranked overall quality of chili-tallow systems.

## 1 Introduction

“Chaotianjiao” (*Capsicum annuum* L. var. *conoides* (Mill*.*) Irish) is one of the most globally significant vegetable crops and culinary ingredients, renowned not only for its pungency but also for its complex aroma profile and rich nutritional value. Its processed product spicy beef tallow hotpot is one of the most popular culinary staples in China and has gradually spread worldwide. The market size of hotpot sales in China's food and beverage industry was projected to reach 668.9 billion yuan by 2025 (Liu, Gao, et al., 2023). The hotpot seasoning is prepared by a complex frying process with beef tallow oil, along with chili peppers, bean paste, and other seasonings (Zhang, Chen, et al., 2025). Chili pepper is the core flavor raw material in beef tallow hotpot seasoning, which serves not only as the primary source of pungency but also as a crucial contributor to the overall volatile flavor profile(R. Zhang et al., 2021). During the thermal processing of chili pepper with beef tallow, Maillard reactions, lipid oxidation, and capsaicinoid thermal degradation occur simultaneously and generate abundant volatiles that define the product's sensory profile. These reactions are mechanistically interconnected: lipid-derived aldehydes participate in Maillard pathways (Zamora & Hidalgo, 2005), while Maillard-derived melanoidins may modulate capsaicinoid oxidative degradation in lipid-rich matrices (Nooshkam et al., 2019). Previous research has demonstrated temperature-dependent changes in carotenoids and capsaicinoids during chili-oil frying(Bai et al., 2023). Since the constituents vary substantially with geographical origin, the origin is expected to be a key determinant of the final quality of chili–beef tallow composites (Zhang, Lu, et al., 2025). Therefore, it is of great importance to investigate how geographical origin differences in chili pepper affect the final quality of chili–tallow hotpot composites.

Current research primarily focuses on manifesting the influence of cultivar varieties and processing techniques on the flavor characterization or individual quality parameters of its products. For example, Li et al. (2024) analyzed chili oil prepared from 10 different chili cultivars using a combination of *E*-nose, GC–MS, and GC-IMS, and found significant differences in color, capsaicin content and volatile components. Yu et al. (2023) also reported that 18 different chili cultivars significantly affect the capsaicin content, color intensity, and volatile compound composition of Sichuan beef tallow hotpot seasoning, and PCA analysis can clearly distinguish seasoning samples corresponding to different cultivars. Furthermore, studies by Zhu et al. (2023) on 7 types of red chili powder and chili oil, and Ye et al. (2020) on fermented chili, all focused on the impact of cultivar differences on the flavor or physicochemical properties of chili processed products.

On the other hand, even for the same variety of agricultural product cultivated in different regions, the soil minerals, altitude, and diurnal temperature fluctuations can markedly affect their biosynthesis and accumulation of key compounds including volatile aroma compounds, pigments, and antioxidants. These origin-related chemical differences in raw materials inevitably propagate through the processing chain and manifest in the final product's quality attributes. Geographical origin has increasingly been recognized as an important determinant of food flavor quality (Huang et al., 2025; Jiang et al., 2021). For example, Huang et al. (2025) demonstrated that geographical origin significantly influenced the flavor characteristics of Qingxiangxing Baijiu and applied an integrated strategy combining descriptive sensory evaluation, GC × GC–MS, *E*-nose, and ICP-MS to discriminate regional differences. This study provides an important methodological precedent for investigating origin-related flavor variation across complex food matrices. Current production of chili-beef tallow composites largely relies on empirical knowledge and traditional practices when selecting chili pepper sources, lacking systematic scientific guidance based on quantitative analysis of origin-related quality differences. Few investigations have systematically examined how geographical origin influences the volatile flavor profiles of chili-tallow hotpot composites. The selection of chili peppers from different origins in practical production requires objective, data-driven guidance to achieve consistent and desirable product quality.

The flavor characterization of food-related products mostly relies on modern analysis techniques. The integration of *E*-nose, GC × GC–MS, and GC-IMS provides complementary advantages for volatile compound analysis in the food domain. E-nose offers rapid, non-destructive screening and pattern recognition of overall aroma profiles(Tian et al., 2026; Y. Wang et al., 2026; X. Zhang et al., 2026). GC–MS delivers reliable identification and quantification of volatile compounds through mass spectral matching(Sun et al., 2020), while GC-IMS enables sensitive detection and visualization of volatile fingerprints, particularly effective for separating isomeric compounds(Xu et al., 2023). This multi-instrument approach ensures comprehensive and accurate characterization of the complex volatile composition compared to single-technique analyses.

Notably, while instrumental analysis techniques can effectively characterize individual quality indicators, a comprehensive evaluation of the overall quality of chili-tallow hotpot composites from different origins remains challenging due to the multidimensional nature of quality attributes (e.g., color, pungency, volatile flavor compounds). The entropy weight-TOPSIS method has emerged as a comprehensive evaluation tool in food quality assessment, as it assigns data-driven weights to multiple evaluation indexes based on entropy values (reflecting information redundancy) and then ranks samples by calculating their relative closeness to the ideal solution(Liu, Xie, et al., 2023, Wang, Fan, Meng and Yang, 2022). This approach reduces subjective influence in weight assignment and enables a holistic comparison of overall quality across samples. Cao et al. (2024) integrated the entropy weight method with grey correlation degree analysis to identify key quality indicators of chicken soup and establish an objective comprehensive evaluation model for distinguishing the quality of traditional, concentrated and blended chicken soup variants. Zhao et al. (2022) adopted the entropy weight method to calculate the comprehensive micronutrient quality index of fruits and vegetables, and combined it with fuzzy recognition method to construct a novel evaluation system for assessing the vitamin and mineral nutritional quality of 24 vegetables and 20 fruits. However, its application in comprehensively assessing the origin-dependent quality of chili-tallow hotpot composites is currently lacking.

To address the aforementioned research gaps and industrial needs, this study systematically compares the flavor quality of chili–tallow systems prepared from chili peppers obtained from six geographical origins using *E*-nose, GC-IMS, GC × GC–MS, and the entropy weight-TOPSIS method. Samples from six distinct geographical origins in China (Kashgar, Wusu, Jingtai, Yumen, Akesu, Ameng) were collected; then the physicochemical indexes (color, capsaicin content) and flavor characteristics of chili-beef tallow hotpot models were comprehensively analyzed. The entropy weight-TOPSIS method was further employed to conduct a holistic evaluation of the overall quality of chili- tallow composites from different origins. This research aims to lay foundation for hot pot base producers in the selection of chili raw materials.

## 2 Materials and methods

### 2.1 Main materials and reagents

#### 2.1.1 Sampling design

Chili peppers of the same cultivar, “Chaotianjiao” (*Capsicum annuum* var. conoides), were obtained from six geographically distinct origins in China: Wusu (WS), Kashgar (KS), Jingtai (JT), Yumen (YM), Aksu (AKS), and Ameng (AM) (Fig. 1). All samples were provided by Sichuan Chuanjiao Seed Technology Co., with the same cultivar and seed source and standardized cultivation and post-harvest procedures. These composite pepper samples were collected from multiple field plots at each origin during the 2024 harvest season. Samples from these plots were subsequently pooled and homogenized to generate one composite sample for each origin. This pooling strategy reduced dependence on any single field plot while maintaining a consistent sampling procedure across the six origins. After harvest, all samples were hot-air dried, with moisture content controlled at approximately 12% before thermal processing with beef tallow.

**Fig. 1:**
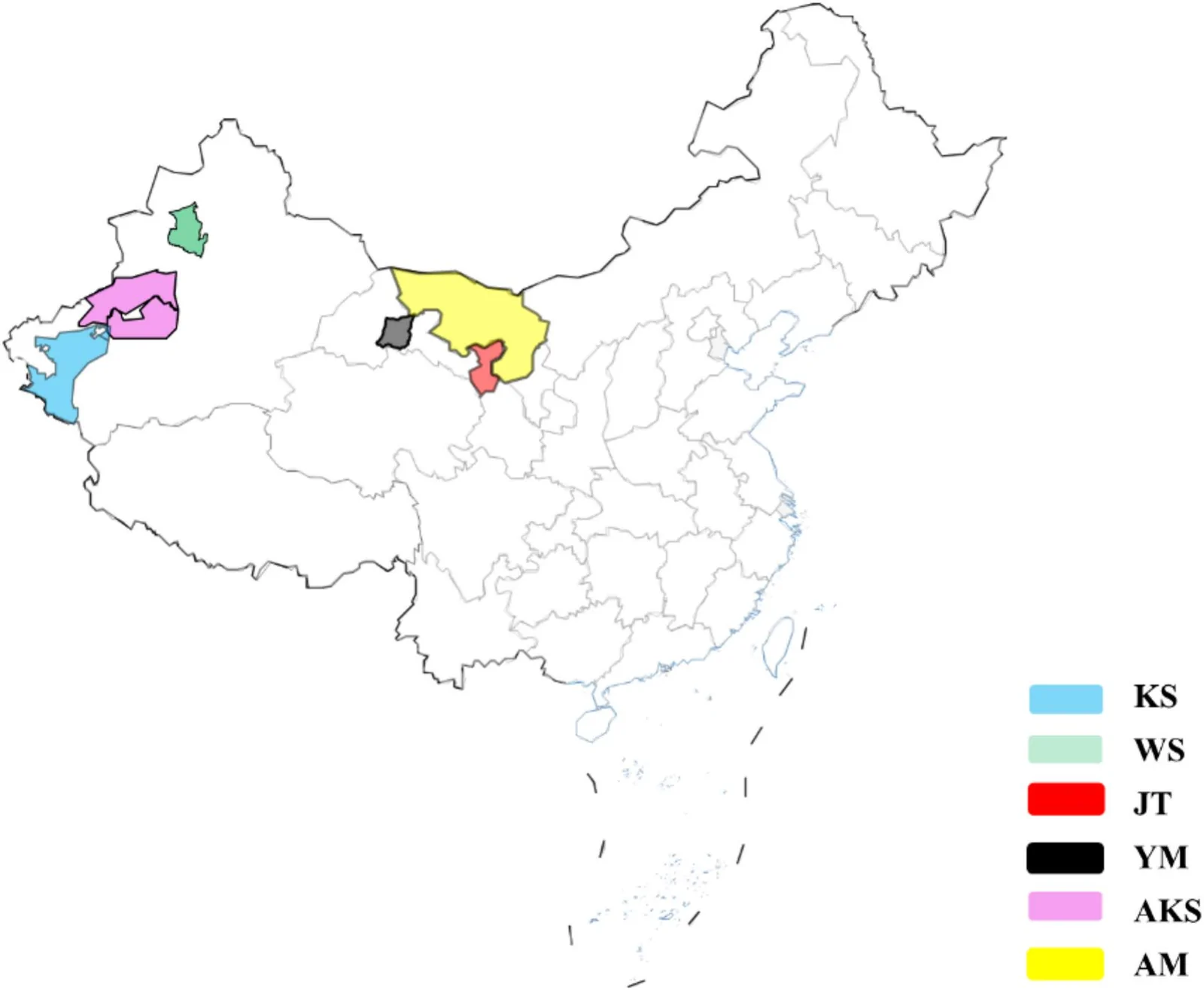
Geographical locations of the six chili pepper origins in China: Wusu (WS), Xinjiang; Kashgar (KS), Xinjiang; Jingtai (JT), Gansu; Yumen (YM), Gansu; Aksu (AKS), Xinjiang; and Ameng (AM), Inner mongolia.

#### 2.1.2 Main reagents

Methanol (99.9%, Thermo Fisher Scientific Inc.), tetrahydrofuran (99.9%, Shanghai Kewel Chemical Technology Co.,Ltd), acetone (99.5%, Thermo Fisher Scientific Inc.), Analytical standards of linear 2-alkanones (C_4_–C_9_), namely 2-butanone, 2-pentanone, 2-hexanone, 2-heptanone, 2-octanone and 2-nonanone (purity ≥99%) and 2,4,5-trimethylthiazole (98%) were obtained from Macklin Biochemical Co., Ltd., Shanghai, China.

### 2.2 Experimental apparatus

The experimental apparatus applied in this study were as follows: FlavourSpec® Gas phase ion mobility spectrometry coupling instrument(Haineng Future Technology Group Co., LTD), gas chromatograph-mass spectrometer(Shimadzu Laboratory Equipment Co., LTD), 75 μm CAR/PDMS fused-silica SPME fiber (Supelco, USA), AIRSENSE-PEN 3 Electronic nose(AIRSENSE Analytics GmbH), Waters e2695 high performance liquid chromatograph (Waters Technology Co., Ltd.), Constant Temperature Water Bath Pot (Harm BoXun Medical Biological Instrument Co., Ltd.), Ultrasonic cleaning machine (Chengdu Century Ark Technology Co., Ltd.), Induction cooker and Crushing Machine (Supor Co., Ltd., China), colorimeter.

### 2.3 Sample pretreatment

Dried chili peppers from each geographical origin were weighed 100 g and added to 1000 mL of cold water at a chili pepper-to-water ratio of 10%, *w*/*v*. The peppers were heated in cold water and boiled for 10 min after the water reached boiling. The boiled peppers were then removed from the water and crushed using a crushing machine to prepare chili sauce. The resulting chili sauce (50 g) was mixed with melted beef tallow at a chili sauce-to-beef tallow ratio of 50%, *w*/w. The mixture was maintained at 120 °C and heated for 70 min. The same preparation procedure and ingredient ratios were applied to chili peppers from all six geographical origins. One chili pepper–beef tallow system was prepared from the composite sample of each origin, giving six origin-associated systems in total. From each system, three samples were taken from different points after thorough mixing and analyzed in parallel as analytical replicates. Pure beef tallow, subjected to the same heating conditions without the addition of chili sauce, was used as the control group (NY).

### 2.4 Sensory evaluation

Sensory evaluation was conducted by nine trained assessors (4 male, 5 female, age 23–34 years), all healthy with no taste or olfactory impairments. Panelists completed 4 h of standardized training on recognizing and rating pungency, aroma, and color. Nine assessors were used for descriptive sensory profiling, and the intensity was evaluated using a 9-point scale (1 = “none”, 9 = “extremely strong”). Evaluations were performed in a white-light sensory room at 22 ± 2 °C. Each 2 g sample was served in clear glass containers labeled with random three-digit codes. Panelists rinsed with purified water between samples. All attributes were rated in duplicate on two separate days, and scores were averaged for analysis. Sensory data were analyzed using one-way repeated-measures ANOVA, with *p* < 0.05 considered significant. The study protocol was approved by the Ethics Committee of Xihua University (approval No. XHLL2025-K005).

### 2.5 Determination of chroma

For each sample, 10 g of the sample were evenly spread in a 5 cm diameter transparent glass petri dish to avoid air bubbles and uneven thickness. Three parallel measurements were conducted at different random positions of each sample, and the average values of lightness (L*), red degree (a*), and yellowness (b*) were recorded. The color difference (ΔE*_ab_) between each chili–beef tallow system and the pure beef tallow control (NY) was calculated using Eq. (1)(Shang et al., 2022):

(1) ΔEab∗=L∗−LNY∗2+a∗−aNY∗2+b∗−bNY∗2#

where NY represents the pure beef tallow control.

### 2.6 Determination of capsaicin and spiciness

Dried pepper samples were ground and sieved to ≤0.425 mm (40-mesh) according to GB/T 21266–2007. A 0.2–0.5 g aliquot (weighed to 0.0001 g) was placed in a 100 mL beaker and extracted with 15 mL of methanol–tetrahydrofuran (1,1, *v*/v). The beaker was sealed with punctured plastic wrap and ultrasonicated at 60 °C for 30 min. After filtration through qualitative filter paper, the residue was re-extracted twice under the same conditions. The filtrates were pooled, concentrated to approximately 30 mL at 70–75 °C under reduced pressure, transferred to a 50 mL volumetric flask, and diluted to volume with the extraction solvent. The solution was filtered through a 0.22 μm nylon membrane and stored at 4 °C in the dark before HPLC analysis.

Capsaicin and dihydrocapsaicin were determined using an LC-16 HPLC system (Shimadzu, Japan) equipped with a ZORBAX SB-C18 column (4.6 mm × 250 mm, 5 μm). The mobile phase consisted of methanol and water (65,35, *v*/v) under isocratic elution at a flow rate of 1.0 mL/min. The column temperature was maintained at 30 °C, the detection wavelength was 280 nm, and the injection volume was 10 μL. Calibration curves were established using capsaicin and dihydrocapsaicin standards. The contents of capsaicin and dihydrocapsaicin were calculated from their respective calibration curves. The total capsaicinoid content (M) was calculated according to GB/T 21266–2007 using Eq. (2), and the pungency level (SHU) was subsequently calculated using Eq. (3).

(2) $$\begin{array}{c} M=\frac{M_{a}+M_{b}}{0.9}\# \end{array}$$

(3) $$\begin{array}{c} \mathrm{SHU}=\frac{M\times 16.1\times 10^{3}}{0.9}+M\times \left(9.3\times 10^{3}\right)\times 0.1\# \end{array}$$

where M represented the total capsaicinoid content (g/kg), *M*_*a*_ was the capsaicin content (g/kg), and *M*_*b*_ was the dihydrocapsaicin content (g/kg). The capsaicinoid content was converted into Scoville Heat Units (SHU) using Eq. (3).

### 2.7 Analysis of volatile flavor substances

#### 2.7.1 *E*-nose analysis

Flavor profiles of chili-beef tallow from six origins were analyzed using a PEN3 electronic nose (Airsense Analytics, Germany) with 10 metal oxide semiconductor sensors: W1C (aromatics), W5S (nitrogen oxides), W3C (aromatic amines), W6S (hydrogen compounds), W5C (aromatic alkanes), W1S (short-chain alkanes), W1W (sulfides, terpenes), W2S (alcohols, aldehydes, ketones), W2W (organic sulfides, aromatics), W3S (long-chain alkanes). The instrument was preheated for 30 min before analysis. For each sample, 3.0 g of homogenized chili-beef tallow was weighed into a 20 mL headspace vial and sealed immediately with a PTFE septum. Vials were incubated at 60 °C for 20 min to equilibrate headspace volatiles. *E*-nose parameters: flush time 125 s, sampling interval 1 s, collection time 120 s, carrier gas (dry air) flow 300 mL/min. Each sample was analyzed in triplicate. Sensor responses (resistance values) were recorded and processed using WinMuster v1.6.2. Radar plots visualized relative sensor response intensities, reflecting the abundance of targeted volatile compound classes.

#### 2.7.2 GC × GC–MS analysis

The sample was melted at 60 °C for 20 min, and 5.0 g was transferred into a 20 mL headspace vial with 1.5 μL of 2,4,5-trimethylthiazole (1.013 μg/mL) as the internal standard(Jin et al., 2022), Individual volatile compounds were semi-quantified using 2,4,5-trimethylthiazole as the single internal standard and expressed as μg/kg of sample, the semi-quantitative expression is presented in Eq. (4). After equilibration at 60 °C for 20 min, headspace volatiles were extracted at 60 °C for 30 min using a 75 μm CAR/PDMS fused-silica SPME fiber (Supelco, USA), followed by splitless thermal desorption at 230 °C for 5 min. GC × GC–MS analysis was performed using a GCMS-QP2020 NX system (Shimadzu, Japan) equipped with an SSM1820 solid-state thermal modulator and an SV modulation column. Separation was achieved using DB-Wax (36.4 m × 0.25 mm × 0.25 μm) and DB-17 ms (1.2 m × 0.18 mm × 0.18 μm) columns with helium at 1.0 mL/min. The oven was held at 40 °C for 3 min, increased to 210 °C at 4 °C/min, and held for 5 min. MS detection was performed in EI full-scan mode with an ion-source temperature of 230 °C, a 3-min solvent delay, a scan range of *m*/*z* 41–330, a scan interval of 0.02 s, and a scan speed of 20,000. Volatile compounds were tentatively annotated by comparison with the NIST 20 mass spectral library, and only compounds with a match factor ≥ 750 were retained for subsequent analysis.

(4) $$\begin{array}{c} C=\frac{A_{1}\left(C_{1}\times v_{1}\right)}{A_{2}\times M}\# \end{array}$$

where *A*_*1*_ and *A*_*2*_ are the peak areas of the analyte and internal standard, respectively, *C*_*1*_ is the internal-standard concentration (μg/mL), *V*_*1*_ is the volume of internal standard added (mL), and *M* is the sample mass (kg).

#### 2.7.3 GC-IMS analysis

Refer to Xu's(Xu et al., 2023) method of GC-IMS and make some minor adjustments. Prior to testing, accurately weigh 2 g of beef tallow sample and transfer it into a 20 mL headspace vial for analysis. Headspace Conditions: Equilibrium temperature: 80 °C; Equilibrium time: 15 min; Headspace injection needle temperature: 85 °C; Injection volume: 500 μL; Carrier gas: Nitrogen; Vial equilibration speed: 500 rpm. GC-IMS Conditions: IMS detector temperature: 45 °C; Chromatographic column: FS-SE-54-CB-1, 15 m, ID: 0.53 mm; Total analysis time: 30 min; Column temperature: 60 °C; Carrier gas: Nitrogen (purity ≥99.99%). Retention indices were calibrated using C4–C9 2-alkanone standards, and volatile compounds were annotated by comparison of retention index and drift time with the GC–IMS database.

#### 2.7.4 Relative odor activity value (rOAV) analysis

Relative odor activity values (rOAVs) were used to estimate the potential aroma contribution of individual volatile compounds. The rOAV for each compound was calculated using Eq. (5). Compounds with rOAV ≥1 were considered potentially important aroma contributors, those with 0.1 ≤ rOAV <1 were considered flavor-modifying compounds, and those with rOAV <0.1 were considered to have a limited aroma contribution(Nie et al., 2024). Therefore, the calculated rOAVs were interpreted as screening indicators of potential aroma contribution rather than absolute measures of sensory impact.

(5) $$\begin{array}{c} \mathrm{rOAVi}=\frac{C_{i}}{T_{i}}\# \end{array}$$

where *C*_*i*_ is the semi-quantitatively estimated content (μg/kg), and *T*_*i*_ represents the threshold of volatile substances. Odor threshold values were obtained from the compilation by van Gemert (2011). When multiple threshold values were available, values reported in oil matrices were preferentially selected to better match the lipid-rich beef tallow system.

#### 2.7.5 Comprehensive evaluation by entropy weight-TOPSIS method

The entropy weight-TOPSIS method combines data-driven weighting with multi-criteria decision-making (Liu, Xie, et al., 2023). This approach reduces subjective influence in weight assignment and enables comprehensive comparison among samples (T. Zhang & Feng, 2024)^.^.The entropy weight–TOPSIS method was used for multivariate comparative evaluation of the chili–beef tallow samples based on the selected physicochemical and aroma-related indicators. Non-detected volatile compounds were assigned a value of zero, and sensitivity analysis was performed by excluding compounds detected in fewer than three origins. TOPSIS was used to calculate the relative closeness of each sample to the ideal solution based on the weighted indicator matrix. The resulting ranking was therefore interpreted as a relative evaluation under the indicator system established in this study rather than as an absolute measure of sensory quality. Indicator weights were calculated using information entropy, and TOPSIS was subsequently used to determine the relative closeness of each sample to the positive and negative ideal solutions. The calculation formula can be referred to Duan ‘s method(Duan et al., 2026). Detailed information is provided in the Supplementary Materials.

### 2.8 Statistical analysis

Origin 2016 (OriginLab Corporation, Northampton, MA, USA) was used to prepare sensory radar plots and correlation heatmaps. Sensory data were analyzed in SPSS 26.0 using one-way repeated-measures ANOVA. Physicochemical and instrumental measurements were performed at least in triplicate. Differences among the six commercial samples were evaluated using one-way ANOVA followed by Duncan's multiple-range test (*p* < 0.05). These comparisons describe the samples analyzed and were not treated as population-level estimates of geographical-origin effects. Pearson correlations were calculated from the six origin-group means (*n* = 6). The analysis examined associations among sensory attributes, color variables, pungency measures, and selected odor-active compounds. PLS-DA was performed in SIMCA 14.1 after Pareto scaling of the GC × GC–MS dataset. Model performance was assessed using R^2^X, R^2^Y, and Q^2^, and robustness was examined with 200 permutations. Variable importance in projection (VIP) scores quantified each volatile's contribution to sample discrimination. Results are reported as mean ± standard deviation.

## 3 Results and discussion

### 3.1 Sensory evaluation

The sensory profiles of the chili–beef tallow systems prepared from chili peppers of different geographical origins are shown in Fig. 2-A. Significant differences among the six geographical origins were observed for all six sensory attributes, including chili aroma, color, fatty, pungency, roasted, and stir-fried (*p* < 0.05; Table S1). Detailed mean scores, ANOVA results, and pairwise comparisons are provided in Table S1.

**Fig. 2:**
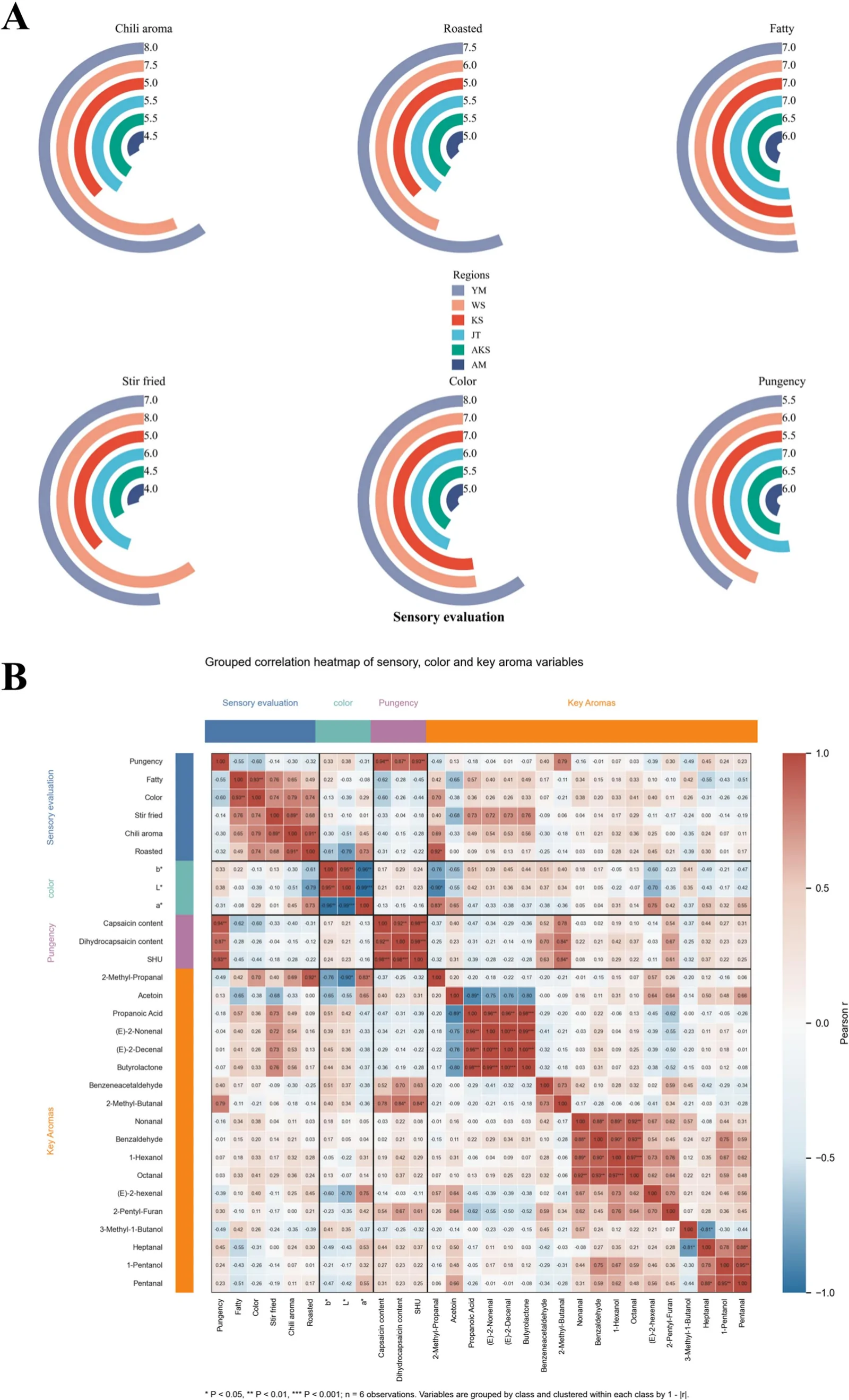
Fig. 2. (A) The influence of different origins on the sensory evaluation of the chili-beef tallow system. (B) Pearson correlation analysis among sensory attributes, color parameters, pungency-related indices, and selected potentially important aroma contributors.

YM exhibited the highest numerical scores for chili aroma, roasted, and color, while WS showed the highest stir-fried score. JT showed the numerically highest pungency score, whereas AM generally exhibited relatively low sensory scores. However, the magnitude of differences between individual geographical origins varied among sensory attributes. Although the overall effects of geographical origin on fatty and pungency were significant, no individual pairwise comparison remained significant after multiplicity correction. Therefore, geographical origin affected the overall sensory profile of the chili–beef tallow systems, but the specific differences among origins depended on the sensory attribute.

To further investigate the relationship between sensory perception and instrumental measurements, Pearson correlation analysis was performed using the mean values of the six geographical-origin groups (*n* = 6). As shown in Fig. 2-B, sensory attributes exhibited different degrees of association with color parameters, pungency-related indices, and selected key odorants. Sensory pungency showed strong positive correlations with capsaicin content (*r* = 0.94), dihydrocapsaicin content (*r* = 0.87), and SHU (*r* = 0.93), indicating a close association between instrumental pungency-related measurements and perceived pungency. In addition, several sensory aroma attributes showed associations with individual key odorants, suggesting that differences in volatile composition may contribute to the sensory variation among geographical origins. Because the correlation analysis was based on six geographical-origin groups, these relationships were considered exploratory associations rather than evidence of causal relationships.

### 3.2 Analysis of chroma

Color characteristics are summarized in Table 1 and visualized in Fig. 3-A. Significant differences in L*, a*, and b* were observed among chili–beef tallow systems from the six origins and pure beef tallow (*p* < 0.05), indicating distinct color characteristics among the samples. L* reflects lightness: pure tallow exhibited the highest L* (35.09), consistent with its pale, luminous appearance; all chili–tallow samples showed lower L*, indicating that capsanthin dissolution into the lipid phase attenuated lightness (Zhou et al., 2021). Among the chili–tallow samples, WS had the highest L* (18.57), whereas JT and YM showed the lowest values (13.41 and 13.37, respectively).

**Table 1:** Color differences of chili-beef tallow samples with different chili producing areas.

Samples	L*	a*	b*	${\mathrm{\Delta E}}_{\mathrm{ab}}^{*}$
KS	14.57 ± 0.73^b^	38.9 ± 1.41^e^	38.26 ± 1.5^e^	49.42
WS	18.57 ± 0.44^d^	31.71 ± 0.37^b^	41.47 ± 0.3^f^	44.34
JT	13.41 ± 0.39^a^	41.13 ± 0.61^f^	27.27 ± 0.56^b^	47.49
YM	13.37 ± 0.75^a^	40.91 ± 1.28^f^	31.4 ± 0.69^c^	48.64
AKS	16.44 ± 0.15^c^	37.03 ± 0.6^d^	35.99 ± 0.81^d^	46.11
AM	17.26 ± 0.13^c^	34.29 ± 0.11^c^	35.84 ± 1.17^d^	43.59
NY(beef tallow)	35.09 ± 0.28^e^	0.89 ± 0.04^a^	14.1 ± 0.15^a^	–

Different lowercase letters of shoulder label in the same column indicated significant difference (*P* < 0.05); −: not applicable; NY was used as the reference sample for ΔE*_ab_ calculation.

**Fig. 3:**
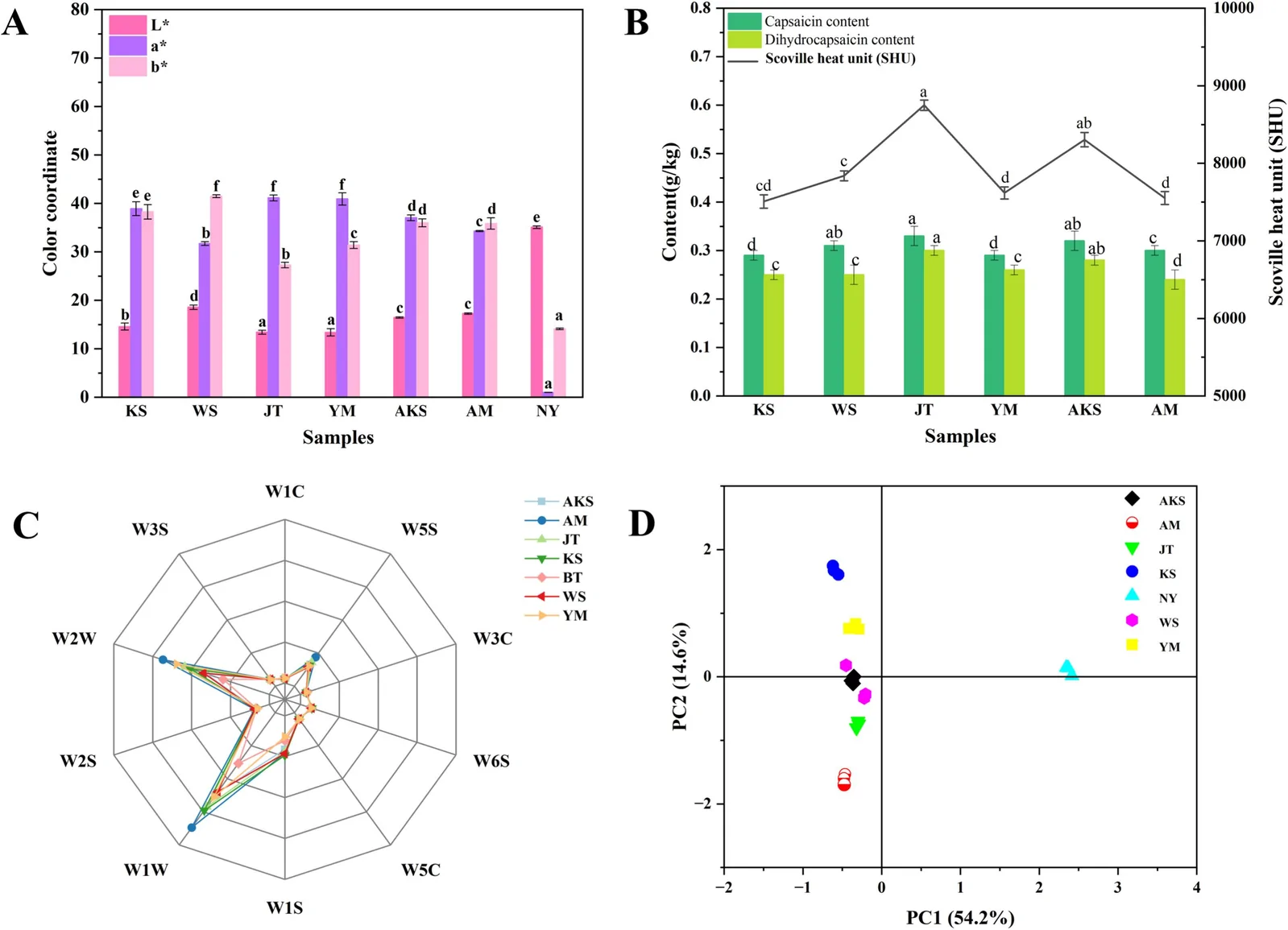
Physicochemical properties and *E*-nose profiles of chili–beef tallow systems from different geographical origins; Different lowercase letters indicate significant differences among the six commercial samples based on technical replicates (*p* < 0.05). (A) Color parameters (L, a, and b*); (B) capsaicin and dihydrocapsaicin contents and Scoville Heat Units (SHU); (C) E-nose sensor response radar plot; (D) PCA score plot based on E-nose responses.

a* represents redness, an important chromatic attribute of hotpot base (Zhang, Chen, et al., 2025). Pure tallow showed a near-zero a* value (0.89), whereas all chili–tallow samples exhibited markedly higher a* values, with JT (41.13) and YM (40.91) showing the highest redness. b* represents yellowness: pure tallow had the lowest b* (14.10), while WS showed the highest value (41.47), followed by KS, AKS, and AM; JT had the lowest b* among the chili–tallow samples (27.27), possibly reflecting differences in pigment composition among origins (Revilla & Quintana, 2005).

Using NY as the reference, the ΔEab values of the chili–beef tallow systems ranged from 43.59 to 49.42. KS showed the greatest color difference from pure beef tallow, whereas AM showed the smallest difference. Overall, the differences in L, a*, b*, and ΔE*ab demonstrated distinct color characteristics of chili–beef tallow systems prepared from different geographical origins.

### 3.3 Analysis of capsaicinoids and pungency

Spiciness is an important flavor of hot pot base, and capsaicin contributed mostly for the spicy taste (Bai et al., 2023). From Table 2 and Fig. 3-B depicted the content of capsaicinoids (capsaicin and dihydrocapsaicin) and the spiciness index in a beef tallow system with the chili from six different origins. In terms of capsaicin, the JT sample had the highest content (0.33 g/kg), followed by AKS (0.32 g/kg) and WS (0.31 g/kg), while KS and YM had the lowest content (both 0.29 g/kg), and AM was at an intermediate level (0.30 g/kg). The trend of dihydrocapsaicin content was similar: JT had the highest content (0.30 g/kg), followed by AKS (0.28 g/kg), while AM had the lowest content (0.24 g/kg), and KS, WS, and YM were at relatively similar intermediate levels (0.24 to 0.26 g/kg). Corresponding to the content of capsaicinoids, the Scoville Heat Units (SHU) - a quantitative indicator of spiciness intensity - showed that JT had the highest spiciness (8749.34), followed by AKS (8304.36) and WS (7839.10), while the SHU values of KS, YM, and AM were relatively low and similar (7508.41 to 7618.14). The differences in capsaicinoid contents among the six commercial samples may reflect variation in preharvest environmental or agronomic conditions, because capsaicinoid accumulation in Capsicum has been reported to respond to factors such as water availability, temperature, and soil conditions(Antonio et al., 2018; Das et al., 2021; Jeeatid et al., 2018).

**Table 2:** The differences in capsaicin content and pungency of chili peppers from different origins in the beef tallow system.

Samples	Capsaicin content(g/kg)	Dihydrocapsaicin content (g/kg)	Scoville heat unit (SHU)
KS	0.29 ± 0.01^d^	0.25 ± 0.01^c^	7508.41 ± 86.43^cd^
WS	0.31 ± 0.01^ab^	0.25 ± 0.02^c^	7839.10 ± 63.41^c^
JT	0.33 ± 0.02^a^	0.30 ± 0.01^a^	8749.34 ± 66.38^a^
YM	0.29 ± 0.01^d^	0.26 ± 0.01^c^	7618.14 ± 77.55^d^
AKS	0.32 ± 0.02^ab^	0.28 ± 0.01^ab^	8304.36 ± 92.34^ab^
AM	0.30 ± 0.01^c^	0.24 ± 0.02^d^	7552.83 ± 83.01^d^

It is noteworthy that the measured values of chroma and pungency are largely consistent with the scoring trends of sensory evaluation, and the two can corroborate each other.

### 3.4 Analysis of volatile flavor substances

#### 3.4.1 *E*-nose analysis

The electronic nose can identify various odors and imitate the human olfactory organ. The electronic nose is highly sensitive to the odor of samples(Hu et al., 2019). Fig. 3-C presents the radar plots of E-nose responses for chili–beef tallow systems. Although all samples exhibit similar radar plot contours—indicating shared volatile compound classes—their sensor response intensities differ markedly. Specifically, responses on W3S, W1C, W5S, W3C, W6S, and W5C sensors converge near baseline (low signal), whereas W1S, W2W, and W1W show consistently higher and discriminative responses across samples.

PCA score plot (Fig. 3-D) reveals clear separation: NY clusters far from all chili–tallow samples along PC1 (54.2% variance), confirming chili addition as the dominant driver of flavor divergence. The six chili–tallow samples (AKS, AM, JT, KS, WS, YM) co-localize in the negative PC1 region but disperse along PC2 (14.6% variance, cumulative 68.8%), indicating that inter-origin flavor differences—though subtler than the chili vs. tallow contrast—are statistically resolvable in multivariate space.

#### 3.4.2 GC × GC–MS analysis

The volatile components in different chili-tallow models were analyzed by GC × GC–MS (Table S2). Meanwhile, a heatmap was drawn based on the content of each volatile component (Fig. 4-A), making the volatile components of the six chili-tallow systems more intuitive. Different colors represent the intensity of the content of different volatile components. The heatmap drawn after normalization shows a color intensity change from the maximum value (reddish-brown) to the minimum value (blue). It can be seen from Table S2 and Fig. 4-A that 81 volatile components were detected in the six chili-tallow samples. Fig. 4-B illustrates the percentage distribution of aldehydes, alcohols, esters, ketones, acids and other substances in different chili-tallow systems. It can be observed in Fig. 4-B that compared with NY, the chili-tallow models with different origin chillies added show varying degrees of increase in alcohols, ketones and acids, while the content of aldehydes in all six chili-tallow models decreased with the addition of chillies.

**Fig. 4:**
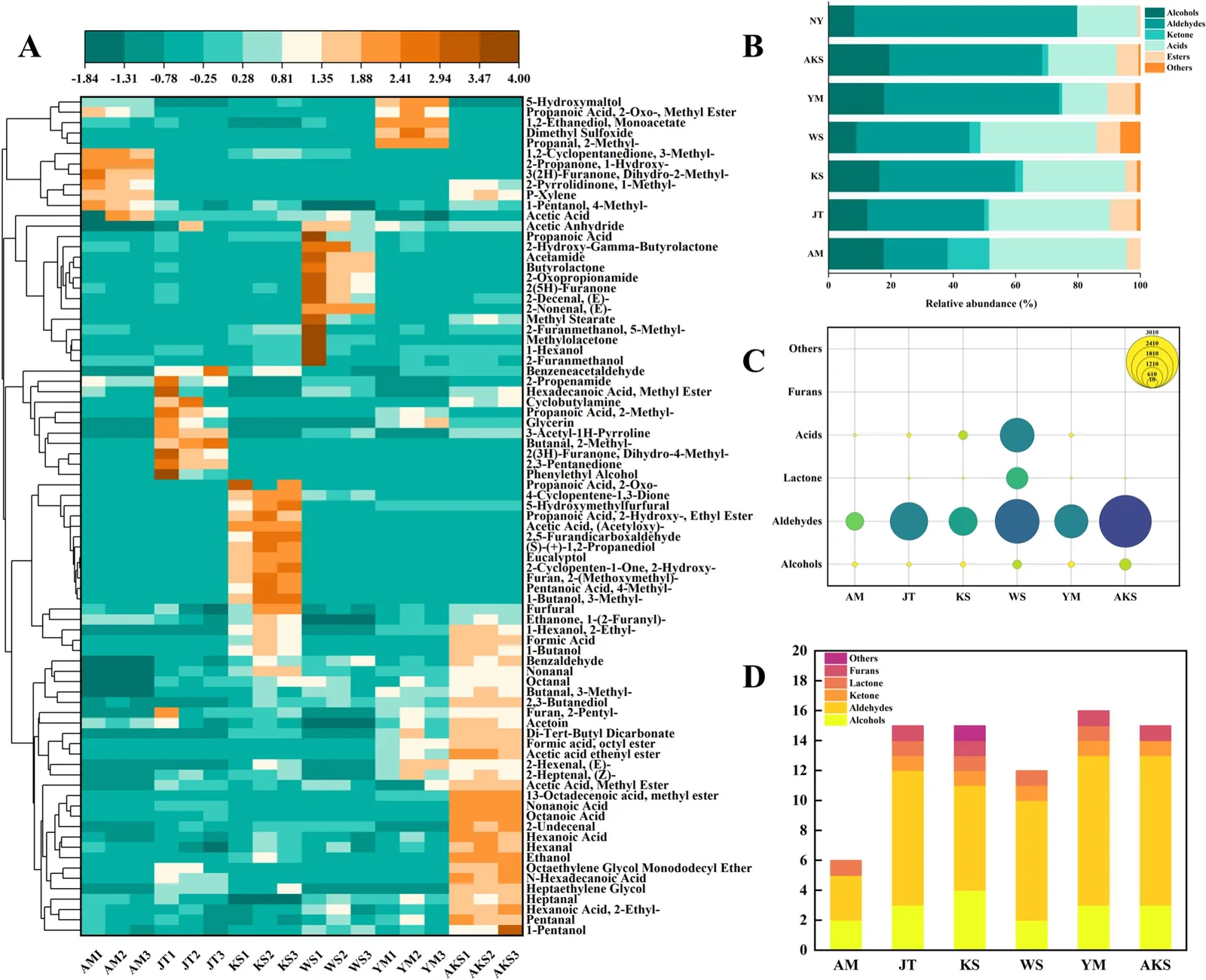
GC × GC–MS volatile profiles of chili–beef tallow systems from different geographical origins. (A) Heatmap of the contents of individual volatile compounds; (B) Relative abundance (%) of different volatile-compound classes in chili–beef tallow systems from different geographical origins.; (C) bubble plot showing the total contents of potentially important aroma contributors (rOAV ≥1) grouped by chemical class, with bubble size representing the summed content; (D) stacked bar chart showing the numbers of potentially important aroma contributors (rOAV ≥1) grouped by chemical class.

##### 3.4.2.1 The influence of the origin of chili peppers on the types and contents of alcohol substances

Alcohols are secondary products of lipid oxidation, mainly derived from the oxidative degradation of unsaturated fatty acids. Their flavor characteristics are closely related to the carbon chain structure and saturation: short-chain fatty alcohols present fresh, sweet and fruity flavors, while medium and long-chain saturated alcohols have soft floral and lipid aromas(Jiang et al., 2021). Unsaturated alcohols (such as linalool) are important contributors to fresh floral and fruity scents and can enhance the perception of sweetness in food(W. Zhu et al., 2019). In this study, The total semi-quantitative content of alcohols varied among the six chili–beef tallow systems, with AKS showing the highest level (4288.9 μg/kg) and WS showing 1778.8 μg/kg, JT showed the lowest (1645.10 μg/kg). 1-Pentanol was present in all six samples, including in pure beef tallow (1124.48 μg/kg). It was observed that the addition of chili peppers significantly reduced the content of 1-pentanol in pure beef tallow. Additionally, 2,3-butanediol, 2-furanmethanol, and 5-methyl-2-furanmethanol were detected in all six samples but not in pure beef tallow; this indicated that the above four substances were specific to chili peppers and did not occur in beef tallow.The influence of the origin of chili peppers on the composition and content of ketone substances.

##### 3.4.2.2 The influence of the origin of chili peppers on the types and contents of ketone substances

Ketone compounds, key contributors to roasted, caramel-like, and creamy aroma notes in thermally processed lipid-based foods(Zou et al., 2018). As shown in Table S2, ketones exhibited distinct distributions among the samples. 2(5H)-Furanone was detected in all chili-added groups, with the highest semi-quantitative content in WS (493.28 μg/kg). Other ketones showed sample-specific occurrence, including dihydro-2-methyl-3(2H)-furanone in AM and 2,3-pentanedione in JT.

##### 3.4.2.3 The influence of the origin of chili peppers on the composition and content of ester substances

In a prior study, Patel et al. (2016) characterized the volatile profiles of 50 native Peruvian fresh chili peppers and reported terpenes, esters, and hydrocarbons as the predominant volatile classes. In contrast, the present thermally processed chili–beef tallow systems showed a greater contribution from aldehydes and other lipid- and heat-derived volatile classes, highlighting the strong influence of matrix and thermal processing on the resulting volatile profile. Significant qualitative and quantitative differences in volatile profiles were observed across the six geographically distinct chili pepper origin groups, as well as in the pure beef tallow control (NY), as summarized in Table S2. In addition, the NY contained only three ester compounds (Hexadecanoic Acid, Methyl Ester; Methyl Stearate; 13-Octadecenoic acid, methyl ester), confirming that endogenous esters in unseasoned beef tallow are limited in both diversity and abundance. The incorporation of chili peppers from different origins drastically altered the ester profile, Methyl acetate: it was detected in JT, KS, WS, YM, and AKS samples, with the highest concentration observed in AKS (808.66 μg/kg). This variation further illustrates the heterogeneous volatile profiles observed among the six commercial chili–beef tallow samples.

##### 3.4.2.4 The influence of the origin of chili peppers on the composition and content of aldehyde substances

Aldehyde compounds, pivotal for green, fatty, and roasted aroma notes in thermally processed foods(W. Li et al., 2026). According to previous research, lipids are initially hydrolyzed into free fatty acids during heating; saturated and unsaturated fatty acids then generate hydrogen peroxides via thermal decomposition, which further contribute to the formation of aldehydes(H. Liu et al., 2019). In comparison with beef tallow, most chili-tallow systems exhibited lower levels of both individual aldehydes and total aldehydes., displayed pronounced quantitative variability and differences among the six commercial samples qualitative distribution across the six chili pepper groups and the pure beef tallow control (NY), as detailed in Table S2. Aldehydes showed pronounced differences in both composition and semi-quantitative content among the chili–beef tallow systems (Table S2). Hexanal was widely detected and showed the highest level among the chili-added samples in AKS (1462.87 μg/kg); however, the NY control contained a substantially higher level (6333.10 μg/kg). Several unsaturated and aromatic aldehydes, including (E)-2-hexenal, benzaldehyde, 2-undecenal, and (E)-2-decenal, showed different occurrence patterns among the six chili-added samples rather than strict origin specificity.

##### 3.4.2.5 The influence of the origin of chili peppers on the composition and content of acidic substances

Acetic acid was detected in all six chili-tallow systems. Gogus et al. (Gogus et al., 2015) found in their research that the production of acetic acid might be due to the slight fermentation of fresh chili during the drying process. In this study, acid compounds showed pronounced variations in composition and concentration across the six chili pepper origin groups and the pure beef tallow control (NY), as presented in Table S2, the NY sample contained four endogenous acids (Propanoic Acid, Acetic Acid, Hexanoic Acid, Octanoic Acid, N-Hexadecanoic Acid), while chili incorporation introduced 11 additional acid derivatives and drastically altered quantitative levels. Acetic acid was detected in all six chili–beef tallow systems, with the highest level in AM (4796.83 μg/kg), followed by WS (4594.24 μg/kg). Propanoic acid varied markedly among samples and reached its highest level in WS (1962.76 μg/kg). The influence of the origin of chili peppers on the composition and content of aldehyde substances.

Taken together, the compositional differences described above among the six chili–beef tallow samples are generally consistent with previous studies showing that chili raw-material variation can substantially affect the physicochemical and volatile profiles of processed chili products. Li et al. (2024) reported cultivar-dependent differences in color, pungency, and volatile composition of chili oils, while Yu et al. (2023) showed that different chili cultivars produced distinct capsaicinoid, sensory, and volatile profiles in Sichuan hotpot oil, with aldehydes, ketones, esters, and acids representing major volatile classes. Y. Zhu et al., 2023 likewise demonstrated marked cultivar-dependent differences in pungency, color, and sensory characteristics of chili powders and oils. Our findings extend these observations by showing measurable variation among commercial samples identified as the same chili cultivar but obtained from different geographical origins. However, the volatile-class pattern observed here differed from that reported for fresh Peruvian peppers by Patel et al. (2016), in which terpenes, esters, and hydrocarbons were among the predominant classes. This discrepancy likely reflects differences in chili germplasm, sample state, food matrix, thermal processing, and analytical conditions rather than a direct contradiction between the studies.

##### 3.4.2.6 Analysis of aroma-active compounds based on rOAV

rOAVs were used to screen volatile compounds with potential contributions to the aroma of the chili–beef tallow systems. As shown in Table 3, 18 compounds exhibited rOAV ≥1 in at least one chili-added sample and were therefore considered potentially important aroma contributors. The number and rOAVs of these compounds varied among the six chili-added samples, with aldehydes and alcohols representing the predominant classes.

**Table 3:** Relative odor activity values (rOAVs) of potentially aroma-active compounds.

	Flavor Descriptors⁎	Threshold (μg/kg)	AM	JT	KS	WS	YM	AKS	NY
**Alcohols**									
1-Pentanol	Alcoholic,Fermented	150.2	0.79 ± 0.17^c^	0.66 ± 0.08^c^	0.73 ± 0.02^c^	1.21 ± 0.09^c^	0.89 ± 0.21^c^	2.16 ± 0.68^b^	7.48 ± 0^a^
1-Hexanol	Mild Sweetness	5.6	–	6.13 ± 1.01^c^	7.97 ± 1.32^c^	6.80 ± 1.38^c^	8.19 ± 2.02^c^	12.52 ± 0.67^b^	48.63 ± 0^a^
Acetoin	Butter Flavor	14	12.33 ± 0.67^b^	8.63 ± 5.08^b^	8.37 ± 1.71^b^	–	12.92 ± 3.4^b^	19.28 ± 0.24^a^	–
3-Methyl-1-Butanol	Alcoholic	6.1	–	–	4.72 ± 0.92^a^	–	–	–	–
**Aldehydes**									
Pentanal	Fermented	850	0.72 ± 0.06^b^	0.61 ± 0.14^b^	0.54 ± 0.11^b^	0.77 ± 0.21^b^	0.85 ± 0.22^b^	1.39 ± 0.05^a^	7.892 ± 0^a^
Heptanal	Green	150	2.1 ± 0.39^c^	1.97 ± 0.58^c^	–	2.29 ± 0.38^c^	2.47 ± 0.55^c^	4.04 ± 0.19^b^	11.36 ± 0^a^
Benzeneacetaldehyde	Hyacinth Fragrance	1.7	63.66 ± 1.41^cd^	292.73 ± 75.65^a^	241.31 ± 49.22^ab^	82.39 ± 13.26^cd^	120.48 ± 30.99^c^	145.53 ± 5.1^bc^	–
2-Methyl-Butanal	Fermented	1.2	–	358.15 ± 79.46^a^	–	–	–	18.54 ± 1.58^b^	–
Octanal	Orange	170	–	0.99 ± 0.09^c^	1.37 ± 0.25^bc^	1.27 ± 0.3^bc^	1.18 ± 0.31^c^	1.77 ± 0.07^b^	3.34 ± 0^a^
Nonanal	Oily	8	–	19 ± 3.23^b^	43.39 ± 6.54^a^	23.29 ± 3.93^b^	24.91 ± 2.67^b^	40.31 ± 1.36^a^	–
(*E*)-2-Nonenal	Fat,Cucumber	0.19	–	78.97 ± 16.87^c^	–	2778.46 ± 65.41^a^	113.83 ± 23.75^c^	202.21 ± 8.85^b^	–
(E)-2-Decenal	Green,Mushrooms	2.7	–	15.79 ± 7.26^c^	9.99 ± 2.88^d^	125.5 ± 62.7^a^	7.94 ± 0.92^e^	17.41 ± 0.84^b^	–
Benzaldehyde	Almond flavor	85	–	0.68 ± 0.09^d^	1.61 ± 0.3^b^	1.63 ± 0.32^b^	0.95 ± 0.06^c^	2.36 ± 0.12^a^	–
(E)-2-hexenal	Cheese flavor	3.1	–	–	6.89 ± 3.04^b^	–	10.75 ± 2.04^a^	10.29 ± 0.7^a^	–
2-Methyl-Propanal	Pungent	1.9	–	–	–	–	39.1 ± 2.12^a^	–	–
**Ketone**									
Butyrolactone	Caramel-like	1000	–	0.14 ± 0.07^b^	0.15 ± 0.02^b^	1.26 ± 0.26^a^	0.13 ± 0.03^b^	0.11 ± 0^b^	–
**Acids**									–
Propanoic Acid	Spicy	1000	0.17 ± 0.07^b^	0.25 ± 0.08^b^	0.52 ± 0.01^b^	1.96 ± 1.2^a^	0.23 ± 0.11^b^	–	0.35 ± 0^b^
**Esters**									
2-Pentyl-Furan	Fruity	19	–	1.53 ± 1^a^	1.13 ± 0.2^a^	–	1.45 ± 0.21^a^	1.96 ± 0.06^a^	–

⁎ :Flavor descriptors are sourced from the website WWW.ChemicalBook.com

Among these compounds, benzeneacetaldehyde was detected in all six chili-added samples and exhibited relatively high rOAVs (63.66–292.73), suggesting an important potential contribution to the floral aroma. (E)-2-Nonenal was detected in JT, WS, YM, and AKS and showed its highest rOAV in WS (2778.46). Other aroma-active aldehydes also showed sample-dependent distributions. For example, (E)-2-decenal was detected in JT, KS, WS, YM, and AKS, whereas (E)-2-hexenal was detected in KS, YM, and AKS.

Several compounds showed more restricted occurrence among the samples examined. 2-Methylbutanal was detected in JT and AKS, with rOAVs of 358.15 and 18.54, respectively; 3-methyl-1-butanol was detected in KS (rOAV = 4.72); and 2-methylpropanal was detected in YM (rOAV = 39.10). Acetoin, with OAVs ranging from 8.37 to 19.28, was detected in AM, JT, KS, YM, and AKS but not in WS. These differences in the occurrence and rOAVs of individual compounds indicate variation in the potential aroma-active profiles among the six commercial chili–beef tallow samples.

Compounds with 0.1 ≤ rOAV <1, including 1-pentanol, pentanal, and butyrolactone, were considered potential flavor-modifying compounds. No compounds with rOAV <0.1 were observed among the compounds included in the rOAV analysis. Overall, aldehydes, particularly benzeneacetaldehyde and (E)-2-nonenal, showed relatively high rOAVs in several samples, while alcohols and other volatile classes may also contribute to the overall aroma profile. Because the rOAVs were calculated using semi-quantitatively estimated contents and literature odor thresholds, these results should be interpreted as screening indicators of potential aroma contribution rather than definitive evidence of sensory impact.

#### 3.4.3 GC–IMS analysis

GC–IMS was employed to characterize the volatile profiles of the chili–beef tallow systems. After standardizing compound nomenclature and consolidating monomeric (M) and dimeric (D) signals corresponding to the same analyte, a total of 44 unique volatile compounds were annotated by GC–IMS (Table S3). These compounds comprised 13 aldehydes, 6 ketones, 5 alcohols, 9 esters, 5 furans, and 6 other compounds. For compounds producing both monomeric and dimeric ion-cluster signals, the M and D signals were retained separately in the fingerprint visualization (Fig. 5A) but were counted as a single chemical compound when calculating the number of unique volatile compounds. Visualized via the 2D GC-IMS fingerprint (Fig. 5-A), pure beef tallow exhibited only a few faint signal spots, indicating limited types and concentrations of endogenous volatile compounds; in stark contrast, samples co-heated with chili pepper showed a dramatic increase in both the number of signal spots and their intensity (reflected by yellow/orange hues for higher concentrations). This observation directly illustrates that chili pepper serves as the dominant source of flavor compounds in the co-heated system, enriching the volatile profile to form the characteristic flavor of the pepper-beef tallow matrix.

**Fig. 5:**
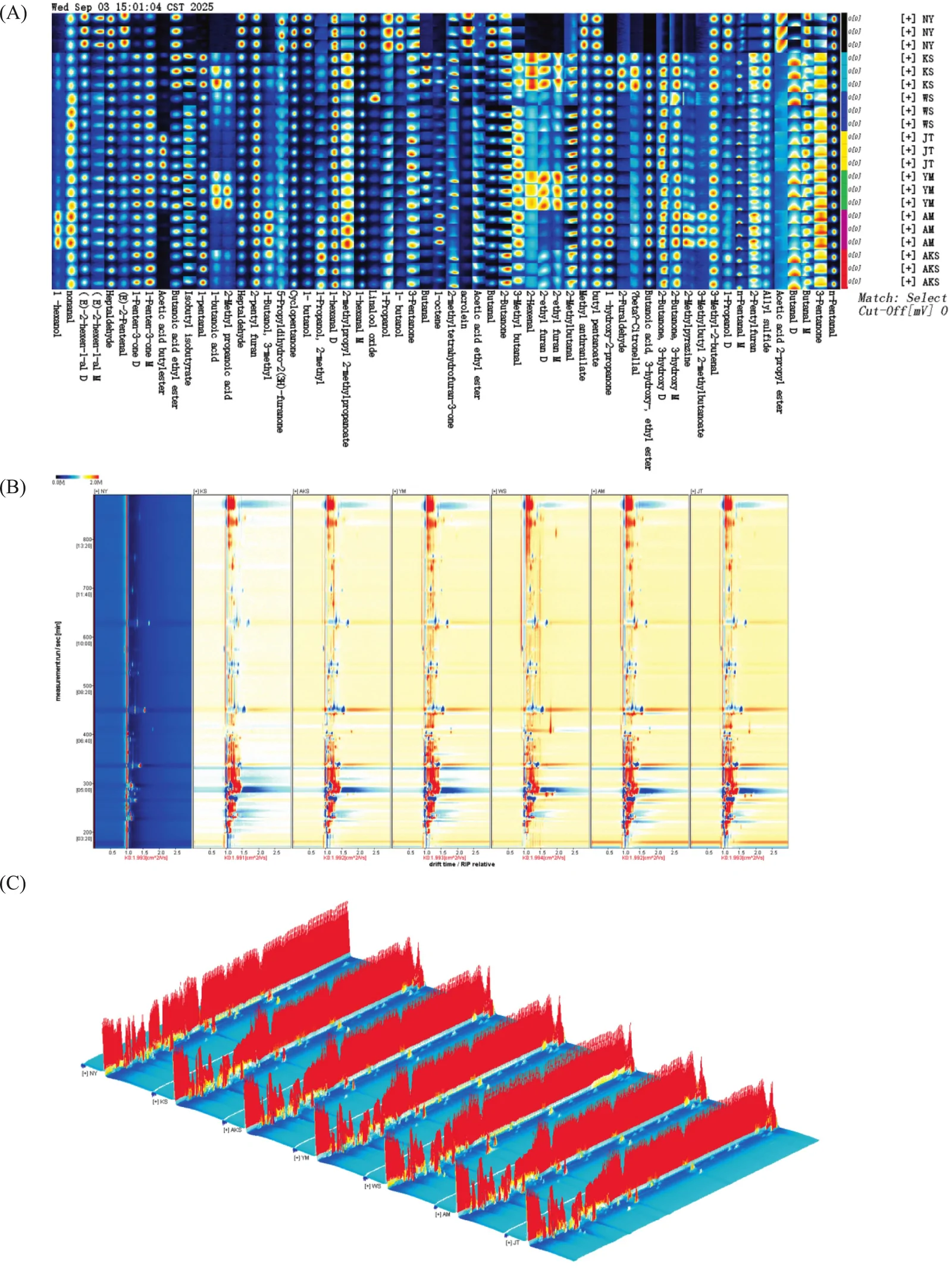
GC–IMS volatile profiles of chili–beef tallow systems from different geographical origins. (A) VOC fingerprint; (B) two-dimensional topographic difference plots; (C) three-dimensional topographic plots. M and D denote monomeric and dimeric ion-cluster signals, respectively; M/D signals originating from the same analyte were counted as one unique compound.

Secondly, to gain a more intuitive understanding of the differences in flavor substances among the various samples, topographic map analysis was conducted on all the chili-tallow samples. Two-dimensional and three-dimensional topographic maps were drawn based on the signal intensity of each compound (Fig. 5-B and 5-C). The results indicated that the signal intensity of all chili-tallow samples was significantly higher than that of the NY, suggesting that the majority of substances in the chili-tallow model were brought by the chili. The types of volatile compounds in the six chili-tallow samples were similar, but the signal intensities were slightly different. To more clearly observe the differences among the samples, the topographic maps of the other samples were subtracted from that of the NY as a reference, resulting in Fig. 5-B. As shown in the figure, the red areas indicate that the concentration of volatile compounds is higher than that of the reference sample, while the blue areas indicate that it is lower. It can be seen that the red areas of the six chili-tallow systems are all higher than that of the NY.

Among the compounds detected by both analytical platforms, 1-butanol, hexanal, pentanal, nonanal, and 1-hexanol were consistently observed in the six chili–beef tallow systems. Their detection by both techniques provides cross-platform support for their occurrence in the volatile profiles of these samples. GC–IMS detected numerous low-molecular-weight ketones and esters, whereas the GC × GC–MS aroma-active subset additionally highlighted higher-chain, unsaturated, and aromatic aldehydes. These patterns demonstrate the complementary analytical coverage of the two platforms. It is worth noting that Pentanal, Nonanal, and 1-Hexanol are also potentially important aroma contributors. These shared components, primarily consisting of aldehydes and alcohols, are key flavor-active compounds derived from lipid oxidation and thermal reactions, and their stable detection across both analytical platforms confirms their essential role in the volatile flavor profile of the chili-tallow systems.

Several volatile compounds were detected by both GC × GC–MS and GC–IMS, whereas differences were also observed between the volatile profiles obtained using the two analytical platforms. These differences likely reflect their distinct separation and detection principles. GC–IMS provides rapid headspace fingerprinting and sensitive detection of highly volatile compounds, whereas GC × GC–MS offers enhanced chromatographic resolution for complex volatile mixtures. Therefore, the two techniques provided complementary information for characterizing the volatile profiles of the chili–beef tallow systems.

#### 3.4.4 PLS-DA analysis

The GC × GC–MS volatile dataset was further subjected to multiclass PLS-DA to explore differences among the six origin-labeled chili–beef tallow samples. Prior to modeling, the data were Pareto-scaled. As shown in Fig. 6A, the six sample groups exhibited clear separation in the PLS-DA score plot, while technical replicate measurements from the same commercial sample clustered relatively closely.

**Fig. 6:**
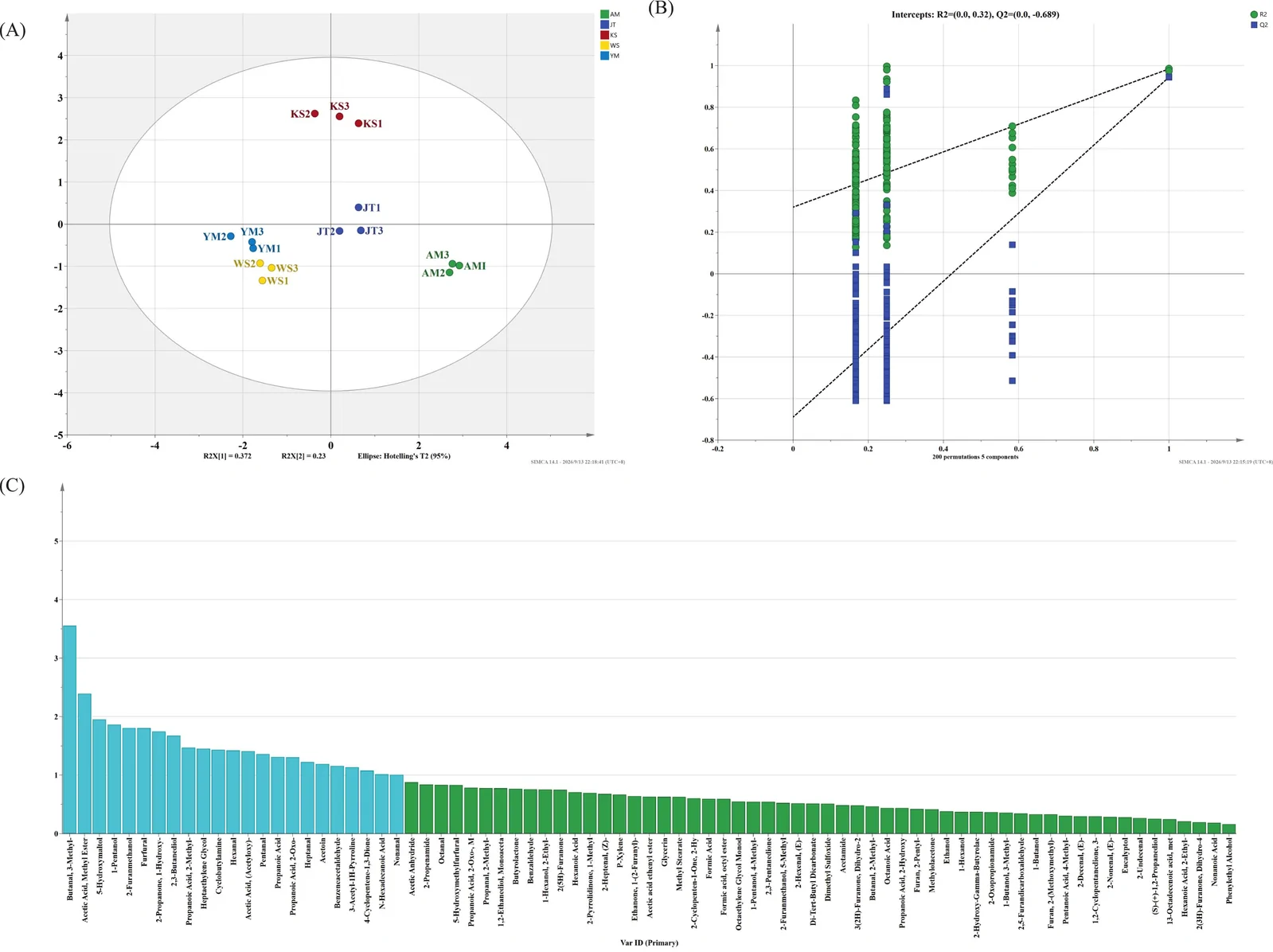
PLS-DA analysis of GC × GC–MS volatile profiles of chili–beef tallow systems from different geographical origins. Model parameters: R^2^X = 0.928, R^2^Y = 0.981, and Q^2^ = 0.954. (A) PLS-DA score scatter plot; (B) 200-permutation test, with intercepts of R^2^ = 0.32 and Q^2^ = −0.689; (C) variable importance in projection (VIP) plot.

The PLS-DA model yielded R^2^X = 0.928, R^2^Y = 0.981, and Q^2^ = 0.954, indicating a high degree of explained variation and internal cross-validated predictability within the present dataset. A 200-permutation test was further performed to evaluate model robustness (Fig. 6B). The permutation-test intercepts were R^2^ = 0.32 and Q^2^ = −0.689, with the permuted models showing markedly lower performance than the original model. These results provided no obvious indication of substantial model overfitting within the present dataset.

VIP analysis was subsequently performed to identify variables contributing most strongly to the observed multivariate separation (Fig. 6C). The highest-ranking VIP variables included 3-methylbutanal, methyl acetate, 5-hydroxymaltol, 1-pentanol, 2-furanmethanol, furfural, 1-hydroxy-2-propanone, 2,3-butanediol, and 2-methylpropanoic acid. These compounds contributed strongly to discrimination among the six samples and may represent candidate variables associated with the observed origin-related differences.

#### 3.4.5 TOPSIS evaluation calculation

In this study, the color values (L*, a*, b*) and potentially important aroma contributors (18 types) were selected as evaluation indicators. A larger b* value indicates a yellower sample color, which is unfavorable for the color of beef tallow hot pot, thus it is regarded as a negative indicator. Color parameters (L, a, and b*) and 18 volatile compounds with rOAV ≥1 were incorporated into the entropy weight–TOPSIS evaluation. The entropy-derived weights reflect the extent to which each indicator differentiates the six samples in the present dataset. Accordingly, the relatively high weights assigned to sparsely distributed compounds, such as 3-methyl-1-butanol and 2-methylpropanal, indicate their strong discriminatory information across the six samples and should not be interpreted as evidence that these compounds are intrinsically more important to sensory quality.

As can be seen from Table 4 and Table 5, after generating weighted data using the entropy weight method, a TOPSIS analysis was conducted. A total of 21 indicators, including color and potentially important aroma contributors, were evaluated by the entropy weight TOPSIS method, with 6 objects being evaluated. The TOPSIS method first determines the positive ideal solution value (R+) and negative ideal solution value (R-) of the evaluation indicators, and then calculates the distance values D+ and D- between each evaluation object and the positive and negative ideal solutions. Based on the D+ and D- values, the proximity (C value) of each evaluation object to the optimal solution is ultimately calculated, and the C values can be ranked. From the ranking results, it can be known that the quality ranking of peppers from the six production areas is as follows: the full-indicator model yielded the ranking YM > WS > KS > JT > AKS > AM. After excluding compounds detected in fewer than three origins, the ranking changed to WS > AKS > YM > KS > JT > AM, indicating that the TOPSIS ranking was sensitive to sparsely distributed volatile indicators (Table S4).

**Table 4:** Summary of the weight calculation results by the entropy value method.

indexes	information entropy value - e	Information utility value - d	Weight coefficient - w
L*	0.7269	0.2731	3.4813%
a*	0.8543	0.1457	1.8573%
b*	0.8296	0.1704	2.1717%
1-Pentanol	0.6263	0.3737	4.7637%
1-Hexanol	0.653	0.347	4.4229%
Acetoin	0.8722	0.1278	1.6290%
3-Methyl-1-Butanol	0.0219	0.9781	12.4673%
Pentanal	0.7336	0.2664	3.3958%
Heptanal	0.878	0.122	1.5554%
Benzeneacetaldehyde	0.7581	0.2419	3.0833%
Butanal， 2-Methyl-	0.1273	0.8727	11.1247%
Octanal	0.889	0.111	1.4151%
Nonanal	0.8707	0.1293	1.6486%
2-Nonenal， (E)-	0.2919	0.7081	9.0258%
(E)-2-Decenal	0.5572	0.4428	5.6443%
Benzaldehyde	0.8523	0.1477	1.8830%
(E)-2-Hexenal	0.6081	0.3919	4.9951%
Propanal， 2-Methyl-	0.0219	0.9781	12.4673%
Butyrolactone	0.5734	0.4266	5.4374%
Propanoic Acid	0.6432	0.3568	4.5479%
Furan， 2-Pentyl-	0.766	0.234	2.9829%

**Table 5:** TOPSIS evaluation calculation results.

Samples	Positive ideal solution distance D+	Negative ideal solution distance D-	Relative proximity C	Sorting result
KS	0.2209	0.1363	0.3816	3
WS	0.2245	0.1427	0.3886	2
JT	0.2326	0.1229	0.3456	4
YM	0.2187	0.1412	0.3922	1
AKS	0.2412	0.0942	0.2808	5
AM	0.264	0.0322	0.1087	6

## 4 Conclusions

This study revealed coordinated differences in odor, volatile composition, color, and pungency among chili–beef tallow systems prepared from origin-associated samples of the same cultivar. Chili quality for beef-tallow hotpot applications is therefore better described by a combination of odor- and color-related indicators than by any single attribute. The TOPSIS ranking was highly sensitive to sparsely distributed volatile indicators: excluding compounds detected in fewer than three origins changed the ranking from YM > WS > KS > JT > AKS > AM to WS > AKS > YM > KS > JT > AM. Volatile profiling further showed that origin-associated flavor differences are chemically resolvable within this dataset. These results indicate that composite rankings based on sparse volatile data are indicator-dependent and should be interpreted with caution, whereas the multidimensional quality differences among origins are directly supported by the measured indicators.

Geographical origin is an integrative rather than a causal variable: because all samples shared the same cultivar and seed source, cultivar-level genotypic variation is unlikely to explain these differences, which more plausibly reflect origin-associated environmental and agronomic factors and possible genotype × environment interactions. As these factors were not independently measured, this attribution remains hypothesis-generating. Because samples from multiple field plots within each origin were pooled into one composite sample, only one independent composite sample was available per origin and within-origin variability could not be estimated. The present findings therefore characterize the six origin-associated composite samples rather than the entire regional populations. Future studies with multiple independent batches and producers per origin, and with controlled cultivation or multi-environment trials, are required to determine whether the origin-associated patterns observed here are reproducible and can support reliable raw-material selection.

## CRediT authorship contribution statement

**Min Xu:** Writing – review & editing, Writing – original draft, Funding acquisition, Data curation, Conceptualization. **Daitian Yan:** Writing – original draft, Software, Investigation, Formal analysis. **Xiaoxiao Chen:** Resources, Conceptualization. **Linjie Hu:** Methodology, Formal analysis, Data curation. **Lan Li:** Methodology, Data curation. **Ling Li:** Methodology, Data curation. **Hongbin Lin:** Resources, Methodology. **Qin Xiang:** Writing – review & editing, Methodology. **Wenwen Hou:** Data curation. **Ping Liu:** Writing – review & editing, Project administration, Funding acquisition, Conceptualization.

## Declaration of competing interest

The authors declare that they have no known competing financial interests or personal relationships that could have appeared to influence the work reported in this paper.

## Data Availability

Data will be made available on request.
