# Supplementary material for: Unraveling the impact of chili pepper geographical origin on beef tallow system: Integrated flavoromics and physicochemical profiling by HS-GC-IMS, GC × GC–MS, and E-nose
Source: Food Chem X. 2026 Oct 26;40:104693. doi: 10.1016/j.fochx.2026.104693 (PMC13742469; doi:10.1016/j.fochx.2026.104693)
Supplement: Supplementary file 1 [file mmc1.docx]

**Supplementary Materials.**

TOPSIS-Entropy Weight Method Calculation Steps

Step 1: Normalize the indicators towards the same trend.

Equation 4 -Positive indicator: The larger the index value, the better the performance of the evaluation object.

Equation 5 -Negative indicator: The smaller the value of the indicator, the better the performance of the evaluation object.

$$\begin{aligned} \text{X}_{\text{ij}}^{\text{`}}\text{=}\frac{\text{X}_{\text{ij}}\text{-}\min\left( \text{X}_{\text{1j}}\text{,X}_{\text{2j}}{\text{,}\text{…}\text{,X}}_{\text{nj}} \right)}{\max\left( \text{X}_{\text{1j}}\text{,X}_{\text{2j}}{\text{,}\text{…}\text{,X}}_{\text{nj}} \right)\text{-}\min\left( \text{X}_{\text{1j}}\text{,X}_{\text{2j}}{\text{,}\text{…}\text{,X}}_{\text{nj}} \right)}\# \left( 5 \right) \end{aligned}$$

$$\begin{aligned} X_{ij}^{`}=\frac{\max\left( X_{1j},X_{2j},\ldots,X_{nj} \right)-X_{ij}}{\max\left( X_{1j},X_{2j},\ldots,X_{nj} \right)-\min\left( X_{1j},X_{2j},\ldots,X_{nj} \right)}\#\left( 6 \right) \end{aligned}$$

In the formula, X_ij_ represents the original values of each indicator, X`_ij_ represents the processed results, max(X_1j_,X_2j_,…,X_nj_) is the maximum value of each indicator, and min(X_1j_,X_2j_,…,X_n_) is the minimum value of each indicator.

Step 2: Construct a non-negative translation matrix - Zj.

Translation of the displacement amount selection: Usually, δ is taken as 0.1 or 0.001. The purpose is to ensure the validity of logarithmic operations: eliminate 0 and negative numbers.

Step 3: Calculate the weights using the entropy weight method.

Step 3.1：Calculate the normalized matrix (P-value).

$$\begin{aligned} P_{ij}=\frac{X_{ij}}{\sum_{i=1}^{n} X_{ij}}\#\left( 7 \right) \end{aligned}$$

Step 3.2：Calculate the information entropy value – e.

$$\begin{aligned} e_{j}=-K\sum_{i=1}^{n} \left( P_{ij}\ln P_{ij} \right)\#\left( 8 \right) \end{aligned}$$

Among them: K = ln(n), where n represents the number of research objects. In this article, n = 6, that is, 6 production areas.step3: Identify the optimal solution vector and the worst solution vector.

Step 3.3：Calculate the information utility value – d.

$$\begin{aligned} d_{j}=1-e_{j}\#\left( 9 \right) \end{aligned}$$

Step 3.4: Calculate the weight coefficient-w.

$$\begin{aligned} w_{j}=\frac{d_{j}}{\sum_{j=1}^{m} d_{j}}\#\left( 10 \right) \end{aligned}$$

Step 4: TOPSIS Comprehensive Evaluation.

Step 4.1: Constructing the weighted matrix – R.

$$\begin{aligned} R_{ij}=w_{j}\cdot Z_{ij}\#\left( 11 \right) \end{aligned}$$

Step 4.2：Determine the positive ideal solution R+ and the negative ideal solution R-.

$$\begin{aligned} R^{+}=max\left( R_{I1},R_{I2},\ldots R_{In} \right)\#\left( 12 \right) \end{aligned}$$

$$\begin{aligned} R^{-}=min\left( R_{I1},R_{I2},\ldots R_{In} \right)\#\left( 13 \right) \end{aligned}$$

Step 4.3: Calculate the distance D+ from the evaluation object to the optimal solution or the distance D- from the worst solution respectively.

$$\begin{aligned} D_{i}^{+}=\sqrt{\sum_{j=1}^{m} Z_{j}\left( R_{IJ}-R_{J}^{+} \right)^{2}}\#\left( 14 \right) \end{aligned}$$

$$\begin{aligned} D_{i}^{-}=\sqrt{\sum_{j=1}^{m} Z_{j}\left( R_{IJ}-R_{J}^{-} \right)^{2}}\#\left( 15 \right) \end{aligned}$$

Step 5: Calculate the value close to program C by combining the distance values and sort them.

$$\begin{aligned} C_{i}=\frac{D_{i}^{-}}{D_{i}^{+}+D_{i}^{-}}\#\left( 16 \right) \end{aligned}$$

**Table S1** Mean sensory scores and statistical analysis of chili–beef tallow systems from different geographical origins

| Attribute | YM | WS | KS | JT | AKS | AM | ANOVA |
| --- | --- | --- | --- | --- | --- | --- | --- |
| Chili aroma | 8.00 ± 0.71 | 7.50 ± 1.00 | 5.00 ± 0.66 | 5.50 ± 1.15 | 5.50 ± 1.20 | 4.50 ± 0.50 | F=20.14, p<0.001 |
| Roasted | 7.50 ± 0.94 | 6.00 ± 0.83 | 5.00 ± 0.83 | 5.50 ± 0.79 | 5.50 ± 0.79 | 5.00 ± 0.94 | F=10.30, p<0.001 |
| Fatty | 7.00 ± 1.12 | 7.00 ± 1.03 | 7.00 ± 0.94 | 6.50 ± 0.71 | 6.00 ± 0.83 | 6.00 ± 0.90 | F=2.73, p=0.032 |
| Stir fried | 7.00 ± 0.97 | 8.00 ± 0.90 | 5.00 ± 1.03 | 6.00 ± 1.17 | 4.50 ± 0.61 | 4.00 ± 1.12 | F=20.40, p<0.001 |
| Color | 8.00 ± 0.94 | 7.00 ± 0.56 | 7.00 ± 0.83 | 6.00 ± 0.75 | 5.50 ± 1.03 | 5.00 ± 1.09 | F=14.27, p<0.001 |
| Pungency | 5.50 ± 0.94 | 6.00 ± 0.79 | 5.50 ± 0.94 | 7.00 ± 0.87 | 6.50 ± 1.15 | 6.00 ± 1.03 | F=3.22, p=0.016 |

**Table S2** Semi-quantitative contents of volatile compounds in chili peppers from different origins after heating with beef tallow.

|  | AM | JT | KS | WS | YM | AKS | NY |
| --- | --- | --- | --- | --- | --- | --- | --- |
| Alcohols |  |  |  |  |  |  |  |
| 1-Pentanol | 119.05±30.99 | 98.98±14.72 | 109.32±4.01 | 181.9±18.02 | 133.41±38.41 | 324.76±123.96 | 1124.78±142.16 |
| 1-Pentanol,4-Methyl- | 79.13±14.39 | 23.37±19.03 | 34.45±8.03 | - | 29.01±7.61 | 44.12±2.5 | - |
| 2,3-Butanediol | 496.13±116.36 | 425.81±59.68 | 961.99±258.93 | 1110.36±169.6 | 1200.29±139.52 | 1994.3±70.97 | - |
| 2-Furanmethanol | 1149.11±252.36 | 555.78±105.79 | 771.02±157.27 | 327.25±67.44 | 695.33±276.14 | 678.77±38.28 | - |
| 5-Methyl-2-Furanmethanol | 45.24±12.22 | 23.35±14.45 | 77.93±17.21 | 121.25±177.57 | 38.55±10.02 | 54.42±3.94 | - |
| 1-Hexanol | - | 34.33±6.94 | 44.64±9.03 | 38.06±7.63 | 45.87±13.86 | 70.12±4.59 | 272.38±21.58 |
| Phenylethyl Alcohol | - | 12.52±13.6 | - | - | - | - | - |
| Glycerin | - | 51.63±26.6 | - | - | 45.52±10.83 | 22.32±1.91 | - |
| Heptaethylene Glycol | - | 298.57±40.22 | 187.4±192.69 | - | - | 499.07±17.46 | - |
| Acetoin | 172.6±11.38 | 120.76±87.02 | 117.14±29.36 | - | 180.91±58.4 | 269.92±4.21 | - |
| Ethanol | - | - | 52.18±38.47 | - | - | 163.04±8.5 | - |
| 1-Butanol | - | - | 24.38±6.43 | - | - | 29.63±2.42 | 393.7±22.07 |
| 3-Methyl-1-Butanol- | - | - | 28.8±6.86 | - | - | - | - |
| 1-Hexanol,2-Ethyl- | - | - | 127.26±16.64 | - | 55.6±11.89 | 138.45±5.19 | - |
| (S)-(+)-1,2-Propanediol | - | - | 11.24±2.89 | - | - | - | - |
| Aldehydes |  |  |  |  |  |  |  |
| Pentanal | 612.89±67.59 | 518.61±142.57 | 459±112.08 | 657.65±211.59 | 725.68±229.81 | 1185.89±57.09 | 6708.2±0 |
| Hexanal | 793.34±83.92 | 583.77±125.27 | 921.85±187.14 | 787.27±206.37 | 728.69±74.03 | 1462.87±75.93 | 6333.1±527.41 |
| Heptanal | 314.87±72.52 | 295.05±106.04 | - | 343.77±70.46 | 370.52±101.01 | 606.31±35.74 | 1704.02±0 |
| Benzeneacetaldehyde | 108.23±2.92 | 497.64±157.51 | 410.22±102.47 | 140.06±27.61 | 204.81±64.54 | 247.4±10.62 | - |
| 2-methylbutanal | - | 429.78±116.77 | - | - | - | 22.24±2.32 | - |
| 3-methylbutanal | - | 1654.37±298.75 | 2634.3±745.6 | 3396.71±958.62 | 4343.16±529.75 | 5266.21±148.07 | - |
| 2-Heptenal,(Z)- | - | 41.29±11.49 | 40.88±8.07 | - | 67.68±20.53 | 76.17±5.14 | - |
| Octanal | - | 168.54±18.57 | 232.08±53.24 | 216.72±62.02 | 200.13±63.65 | 300.51±13.81 | 569.17±34.32 |
| Nonanal | - | 152.01±31.68 | 347.11±64.11 | 186.31±38.53 | 199.3±26.1 | 322.44±13.34 | - |
| (E)-2-Nonenal | - | 15±3.93 | - | 527.91±15.22 | 21.63±5.53 | 38.42±2.06 | - |
| (E)-2-Decenal | - | 42.62±24.03 | 26.98±9.53 | 338.84±207.33 | 21.43±3.06 | 47±2.78 | - |
| 2-Undecenal | - | 4.22±1.4 | 5.33±0.86 | 8.16±2.1 | - | 25.82±3.31 | - |
| Benzaldehyde | - | 57.52±9.62 | 137.05±31.62 | 138.92±33.65 | 80.82±5.64 | 200.71±12.92 | - |
| (E)-2-hexenal | - | - | 21.35±11.54 | - | 33.32±7.75 | 31.89±2.67 | - |
| Furfural | 559.27±126.14 | 476.34±344.02 | 1146.49±254.99 | 354.48±113.21 | 564.42±162.63 | 873.55±26.18 | - |
| 4-Cyclopentene-1,3-dione | - | - | 236.09±40.72 | 82.79±5.32 | - | - | - |
| 5-Hydroxymethylfurfural | - | - | 165.14±46.05 | - | 33.92±6.37 | 37.13±1.91 | - |
| Ketone |  |  |  |  |  |  |  |
| Dihydro-2-Methyl-3(2H)-Furanone | 68.98±18.88 | - | - | - | - | - | - |
| Methylolacetone | - | - | 46.54±25.01 | 196.35±318.74 | - | - | - |
| 1-hydroxy-2-Propanone | 985.62±24.06 | - | - | - | - | - | - |
| 1-(2-Furanyl)-Ethanone | 110.94±5.13 | 59.74±18.83 | 143.75±27.9 | - | 63.18±22.81 | 122.92±4.18 | - |
| 2(5H)-Furanone | 95.35±23.51 | 73.91±40.29 | 99.74±24.52 | 493.28±202.73 | 70.08±20.5 | 88.55±3.62 | - |
| 1,2-Cyclopentanedione,3-Methyl- | 24.12±4.36 | - | 10.83±2.53 | 5.33±1.68 | - | - | - |
| 2,3-Pentanedione | - | 52.29±13.65 | - | - | - | - | - |
| 2-Cyclopenten-1-One,2-Hydroxy- | - | - | 89.54±14.72 | - | - | - | - |
| 2(3H)-Furanone,Dihydro-4-Methyl- | - | 8.16±4.54 | - | - | - | - | - |
| P-Xylene | 128.8±3.18 | - | - | - | - | 124.31±3.34 | - |
| 2-Pyrrolidinone,1-Methyl- | 141.62±36.36 | - | - | - | - | 87.64±2.58 | - |
| Acids |  |  |  |  |  |  |  |
| Propanoic Acid | 170.33±85.15 | 251.98±105.91 | 519.58±18.86 | 1962.76±1472.03 | 233.26±135.97 | - | 349.49±0 |
| Acetic Acid | 4796.83±452.87 | 3451.77±256.38 | 3469.89±578.89 | 4594.24±569.49 | 844.83±169.49 | 2577.72±58.24 | 2471.44±152.43 |
| Hexanoic Acid | 38.9±15.05 | 43.25±12.1 | 65.15±14.48 | 50.06±11.39 | 63.44±12.51 | 139.83±5.95 | 232.24±2.93 |
| Hexanoic Acid,2-Ethyl- | 54.84±8.08 | 51.39±19.77 | 42.9±7.8 | 75.86±31.2 | 62.4±11.56 | 133.72±5.12 | - |
| 2-Methylpropanoic Acid | - | 355.34±108.43 | - | - | 207.61±61.99 | - | - |
| Octanoic Acid | - | 32.44±22.17 | 9.04±3.88 | - | 12.71±5.12 | 226.15±6.3 | 132.94±0 |
| Nonanoic Acid | - | 6.75±2.45 | - | - | - | 34.1±2.67 | - |
| N-Hexadecanoic Acid | - | 620.41±88.17 | - | 142.99±63.26 | 60.29±5.63 | 1202.26±26.66 | 955.05±0 |
| Formic Acid | - | - | 67.46±15.57 | - | - | 86.36±5.04 | - |
| Propanoic Acid,2-Oxo- | - | - | 291.94±260.69 | - | - | - | - |
| Pentanoic Acid,4-Methyl- | - | - | 21.27±5.9 | - | - | - | - |
| Propanoic Acid,2-Oxo-,Methyl | 51.91±11.43 | - | - | - | 67.84±14.82 | - | - |
| Acetic Anhydride | - | 297.34±266.71 | 188.14±14.63 | 546.7±141.4 | 411.05±131.37 | 394.94±13.83 | - |
| Acetyloxyacetic acid | - | - | 435.65±0.03 | - | - | - | - |
| Esters |  |  |  |  |  |  |  |
| 1,2-Ethanediol,Monoacetate | 28.45±12.32 | 18.37±10.07 | - | 20.31±9.52 | 104.86±2.39 | 13.06±1.69 | - |
| Hexadecanoic Acid,Methyl Ester | 21.39±7.98 | 39.54±36.46 | 8.01±1.08 | 30.56±7.62 | 7.92±0.57 | 39.26±2.84 | 113.95±0 |
| Methyl acetate | - | 421.44±77.64 | 281.6±58.16 | 390.93±122.21 | 273.49±337.6 | 808.66±33.34 | - |
| Propanoic Acid,2-Hydroxy-,Ethyl Ester | - | - | 42.88±9.75 | - | - | - | - |
| Methyl Stearate | - | - | - | 31.84±29.8 | - | 20.42±2.47 | 62.63±7.18 |
| Acetic acid ethenyl ester | - | - | - | - | 50.6±13.5 | 100.06±4.61 | - |
| Formic acid,octyl ester | - | - | - | - | 44.86±9.95 | 65.99±3.71 | - |
| 13-Octadecenoic acid,methyl ester | - | - | - | - | 7.65±1.55 | 53±3.17 | - |
| 2-Hydroxy-Gamma-Butyrolactone | 55.22±11.34 | 30.78±20.02 | 36.03±3.92 | 198.3±81.06 | 39.99±17.89 | 26.81±2.05 | - |
| 2-Pentyl-Furan | - | 29±23.2 | 21.54±4.65 | - | 27.46±4.83 | 37.25±1.39 | - |
| 5-Hydroxymaltol | 264.24±27.43 | - | 109.64±30.25 | 60.39±26.02 | 582.28±20.71 | - | - |
| Furan,2-(Methoxymethyl)- | - | - | 23.93±4.02 | - | - | - | - |
| 2,5-Furandicarboxaldehyde | - | - | 26.91±8.62 | - | - | - | - |
| 2-Propenamide | 131.27±29.84 | 178.88±67.88 | - | 75.2±12.39 | - | 51.29±2.61 | - |
| Cyclobutylamine | - | 39.63±33.74 | - | - | - | 28.6±1.88 | - |
| Octaethylene Glycol Monododecyl Ether | - | 43.69±37.34 | - | - | - | 91.17±3.91 | - |
| 3-Acetyl-1H-Pyrroline | - | 328.35±43.42 | - | 125.35±28.39 | - | 158.04±5.73 | - |
| Eucalyptol | - | - | 17.41±2.68 | - | - | - | - |
| Di-Tert-Butyl Dicarbonate | - | - | 14.7±4.21 | - | 29.75±13.21 | 47.42±2.55 | - |
| Dimethyl Sulfoxide | - | - | - | - | 39.09±10.31 | - | - |
| 2-Oxopropionamide | - | - | - | 181.53±81.44 | - | - | - |
| Acetamide | - | - | - | 380.05±112.29 | - | - | - |
| Others |  |  |  |  |  |  |  |
| 2-Methyl-Propanal | - | - | - | - | 74.28±4.93 | - | - |
| Butyrolactone | - | 138.28±88.57 | 148.84±29.91 | 1253.89±316.07 | 130.54±42.36 | 112.02±6.35 | - |

**Table S3.** Standardized list of unique volatile compounds annotated by GC–IMS in chili–beef tallow systems.

|  | Standardized compound | Signal form | Class |
| --- | --- | --- | --- |
| 1 | Pentanal | Unlabeled | Aldehydes |
| 2 | 3-Pentanone | Unlabeled | Ketones |
| 3 | Butanal | M | Aldehydes |
| 4 | Butanal | D | Aldehydes |
| 5 | Acetic acid 2-propyl ester | Unlabeled | Esters |
| 6 | Allyl sulfide | Unlabeled | Others |
| 7 | 2-Pentylfuran | Unlabeled | Furans |
| 8 | Pentanal | Unlabeled | Aldehydes |
| 9 | 1-Propanol | Unlabeled | Alcohols |
| 10 | 3-Methyl-2-butenal | Unlabeled | Aldehydes |
| 11 | 3-Methylbutyl 2-methylbutanoate | Unlabeled | Esters |
| 12 | 2-Methylpyrazine | Unlabeled | Others |
| 13 | 3-Hydroxy-2-butanone | M | Ketones |
| 14 | 3-Hydroxy-2-butanone | D | Ketones |
| 15 | Ethyl 3-hydroxybutanoate | Unlabeled | Esters |
| 16 | β-Citronellal | Unlabeled | Aldehydes |
| 17 | 2-Furaldehyde | Unlabeled | Furans |
| 18 | 1-Hydroxy-2-propanone | Unlabeled | Ketones |
| 19 | Butyl pentanoate | Unlabeled | Esters |
| 20 | Methyl anthranilate | Unlabeled | Esters |
| 21 | 2-Ethylbutanal | Unlabeled | Aldehydes |
| 22 | 2-Ethylfuran | M | Furans |
| 23 | 2-Ethylfuran | D | Furans |
| 24 | 2-Hexenal | Unlabeled | Aldehydes |
| 25 | 3-Methylbutanal | Unlabeled | Aldehydes |
| 26 | 2-Butanone | Unlabeled | Ketones |
| 27 | Butanal | Unlabeled | Aldehydes |
| 28 | Ethyl acetate | Unlabeled | Esters |
| 29 | Acrolein | Unlabeled | Aldehydes |
| 30 | 2-Methyltetrahydrofuran-3-one | Unlabeled | Furans |
| 31 | 1-Octene | Unlabeled | Others |
| 32 | Butanal | Unlabeled | Aldehydes |
| 33 | 3-Pentanone | Unlabeled | Ketones |
| 34 | 1-Butanol | Unlabeled | Alcohols |
| 35 | 1-Propanol | Unlabeled | Alcohols |
| 36 | Linalool oxide | Unlabeled | Others |
| 37 | Hexanal | M | Aldehydes |
| 38 | Isobutyl isobutyrate | Unlabeled | Esters |
| 39 | Hexanal | D | Aldehydes |
| 40 | 2-Methyl-1-propanol | Unlabeled | Alcohols |
| 41 | 1-Butanol | Unlabeled | Alcohols |
| 42 | Cyclopentanone | Unlabeled | Ketones |
| 43 | 5-Propyldihydro-2(3H)-furanone | Unlabeled | Furans |
| 44 | 3-Methyl-1-butanol | Unlabeled | Alcohols |
| 45 | 2-Pentylfuran | Unlabeled | Furans |
| 46 | Heptanal | Unlabeled | Aldehydes |
| 47 | 2-Methylpropanoic acid | Unlabeled | Others |
| 48 | Butanoic acid | Unlabeled | Others |
| 49 | Pentanal | Unlabeled | Aldehydes |
| 50 | Isobutyl isobutyrate | Unlabeled | Esters |
| 51 | Ethyl butanoate | Unlabeled | Esters |
| 52 | Butyl acetate | Unlabeled | Esters |
| 53 | 1-Penten-3-one | M | Ketones |
| 54 | 1-Penten-3-one | D | Ketones |
| 55 | (E)-2-Pentenal | Unlabeled | Aldehydes |
| 56 | Heptanal | Unlabeled | Aldehydes |
| 57 | (E)-2-Hexenal | M | Aldehydes |
| 58 | (E)-2-Hexenal | D | Aldehydes |
| 59 | Nonanal | Unlabeled | Aldehydes |
| 60 | 1-Hexanol | Unlabeled | Alcohols |

M and D denote monomeric and dimeric ion-cluster signals, respectively. M and D signals assigned to the same analyte were treated as one unique volatile compound for compound-count statistics. Duplicate entries and synonymous compound names were standardized and consolidated before counting.

**Table S4.** Sensitivity analysis of the entropy weight–TOPSIS evaluation after excluding volatile compounds detected in fewer than three geographical origins.

| Samples | Positive ideal solution distance D+ | Negative ideal solution distance D- | Relative proximity C | Sorting result |
| --- | --- | --- | --- | --- |
| KS | 0.225885105 | 0.086193314 | 0.276191203 | 4 |
| WS | 0.130276679 | 0.223125132 | 0.631363862 | 1 |
| JT | 0.237334333 | 0.08152592 | 0.255679155 | 5 |
| YM | 0.220749232 | 0.103533744 | 0.319269747 | 3 |
| AKS | 0.197478904 | 0.147006459 | 0.42674225 | 2 |
| AM | 0.253406423 | 0.050367152 | 0.165804916 | 6 |

**Original data of capsaicin and dihydrocapsaicin, along with the plotted standard curve**

| Concentration (μg/mL) | Capsaicin | dihydrocapsaicin |
| --- | --- | --- |
| 0 | 0 | 0 |
| 10 | 86155 | 85520 |
| 20 | 146500 | 145104 |
| 40 | 267180 | 263833 |
| 60 | 382937 | 390926 |
| 80 | 503630 | 503207 |

capsaicin

| Concentration (μg/ml) | Peak area |
| --- | --- |
| 10 | 86155 |
| 20 | 146500 |
| 40 | 267180 |
| 60 | 382937 |
| 80 | 503630 |
| 100 | 604822 |

dihydrocapsaicin

| Concentration (μg/ml) | Peak area |
| --- | --- |
| 10 | 85520 |
| 20 | 145104 |
| 40 | 263833 |
| 60 | 390926 |
| 80 | 503207 |
| 100 | 615226 |
